# Preventing and fighting stigma: a lesson from the first Mpox in Veneto region of Northeast Italy—A case report

**DOI:** 10.3389/fpubh.2023.1141742

**Published:** 2023-05-19

**Authors:** Tatjana Baldovin, Gloria Girolametto, Ruggero Geppini, Matteo Bordignon, Mauro Alaibac

**Affiliations:** ^1^Department of Cardiac, Thoracic, Vascular Sciences and Public Health, Unit of Hygiene and Public Health, University of Padua, Padua, Italy; ^2^Unit of Dermatology, University of Padua, Padua, Italy

**Keywords:** Mpox, monkeypox, case report, prevention, stigma

## Abstract

Since the 1970s, human monkeypox (Mpox) has been referred to as a zoonotic endemic disease of specific regions of Africa until early 2022, when a worldwide epidemic outbreak developed. There are many hypotheses on how Mpox could spread to non-endemic regions; the dominant theory is that it spread from the UK and Spain among men who have sex with men (MSM). Therefore, the first clinical case in the Veneto region (Northeast of Italy) was analyzed—which represented a typical case report of the ongoing outbreak—with lesions located mainly in the areas associated with sexual behaviors (genital and oral). This case report highlights the new challenges of Mpox, as it seems to differ from the previous classic manifestation. Indeed, although the patient achieved restitution *ad integrum* of lesions and complete recovery from the disease, it is deemed necessary to offer communication strategies to involve a heterogeneous audience based on different risks of exposure but without stigmatizing attitudes, avoiding the mistakes made with HIV. The need for broad public involvement is demonstrated by identifying Mpox even in “anomalous cases.” Stigma could be an obstacle in engaging patients in proper care and in getting honest answers while contact tracing, as happened in our patient's case; thus, WHO recently renamed monkeypox as Mpox. Abnormal outbreaks in non-endemic countries, with no causal links, must become a warning signal for governments and health policies to design national plans for managing unexpected outbreaks. For an effective public health response, health institutions must communicate effectively, focus on changes and prevention measures, and formulate a plan based on equity and inclusion of the most vulnerable groups.

## 1. Introduction

Mpox virus (monkeypox virus or MPXV) is an orthopoxvirus and the causative agent of a viral zoonosis that can spread from animals to humans or from person to person or, incidentally, can infect a person if in close contact with contaminated material (1). The Mpox virus was first isolated and identified in 1958 when a group of monkeys manifested a smallpox-like disease (2). Outside Africa, from 1958 to 1970, eight outbreaks were identified in the United States and the Netherlands among groups of captive monkeys imported from Eastern countries (3). Until 1970, natural outbreaks were detected only in animals (3). The first confirmed human case of monkeypox (MPX) infection, recently renamed by WHO as Mpox, was reported in 1970 when the virus was isolated in a 9-month-old boy admitted to a hospital in the Democratic Republic of the Congo (DRC) (3, 4). To date, the Mpox virus has been considered endemic in the rural rainforests of West and Central Africa, where it has frequently been responsible for sporadic human cases and epidemics (5). Simultaneous with the universal eradication of smallpox, Mpox began to affect diverse populations globally (6). From early on, multiple international travelers' cases have been described with related sporadic clusters outside of Africa (5). Across the world, since the virus was identified and isolated, without considering the 2022 epidemic, more than 35,000 cases (including confirmed, suspected, and probable ones) were documented, of which more than 95% have been reported from the DRC alone, followed by Nigeria and other neighboring African countries (7–9). In the early stages, during the 1970s, coincident immunity to the Mpox virus was achieved with vaccinia vaccination. However, with the eradication of smallpox and the subsequent lack of vaccination efforts, the way was open for the Mpox virus to regain clinical relevance (8).

In 2017, West Africa's largest outbreak occurred in Nigeria after years of no identified cases (10). Months later, Mpox was reported in travelers from Nigeria to Israel, the United Kingdom, Singapore, and the United States of America between September 2018 and November 2021 (11–13). As of May 2022, hundreds of Mpox cases were identified in multiple non-endemic countries, bringing attention to the disease and leading the WHO to report the beginning of the largest outbreak ever recorded outside Africa, with 780 laboratory-confirmed cases by 27 European and North American countries (14). These cases include the first case from the Veneto region of Northeast Italy that we analyzed, which represents a typical case of the ongoing outbreak, with lesions located mainly in sexual areas (genital and oral). This case report highlights the new medical challenges facing Mpox, as it appears to differ from the previous classic manifestation. This was the first time that cases and sustained chains of transmission had been reported in countries without direct or immediate epidemiological links to the regions of Western Africa or Central Africa (15–17). Although there may have been times of undetected transmission (17), nevertheless, this outbreak could represent a highly unusual event linked to a hidden causal nexus (18, 19).

Mpox cases continued to increase in later months. As of 16 March 2023, WHO reported 86,496 laboratory-confirmed cases worldwide in 110 States, with 111 deaths, most of which occurred because of HIV infection or other immunocompromised conditions that were untreated or uncontrolled (20). WHO reported that, less than a year after the outbreak began, the epidemic shows a long-tail epidemic curve with a slow decline in the number of cases. Over the past 3 weeks prior to 16 March 2023, the American countries, which account for 92% of the reported cases, and Western Pacific regions have been experiencing the most significant increases when compared with the other regions, including Europe, where there has been a decrease in Mpox cases. Based on available information, young males between 29 and 41 years of age have been disproportionally affected by this outbreak, as the 96.4% of cases have been reported between them (20). Those affected by the current outbreaks tend to be gay, bisexual, or other men who have sex with men (21, 22). In Europe, the risk of spreading the disease to people who frequently have multiple sexual partners, such as groups of men who have sex with men (MSM), is high to moderate (15, 23, 24). In contrast, the risk of Mpox spreading to the rest of the population who does not have more than one sexual partner is considered low (15, 23).

### 1.1. Transmission and risk factors

Human-to-human transmission could result from close contact with respiratory secretions (droplets) that usually require prolonged face-to-face contact, skin lesions of an infected person, or recently contaminated objects (13). Based on the epidemiological parameters of the disease, until 2019, Mpox was assumed to be unable to generate significant epidemics in human populations without a proximate source of the infected animal population (25). Sexual transmission of the Mpox virus has never been demonstrated until 2022. Still, it has been hypothesized based on previous reports of vaccinia virus transmission through sexual intercourse and the high rate of genital lesions (68%) observed among Mpox cases in Nigeria (5, 26–28). Data collected from the new outbreak, which began in May 2022, suggested that the cause of the Mpox virus infection was due to human-to-human sexual transmission (24, 29).

### 1.2. Signs and symptoms

Mpox is usually a self-limited disease with symptoms lasting 2–4 weeks. In recent decades, the Mpox case fatality ratio has been shown to be around 3–6% (13). The incubation period is 6–13 days (13). The manifestations of the infection begin with the invasion period (0–5 days) characterized by fever, intense headache, back pain, myalgia, intense asthenia, and lymphadenopathy (30), which is a distinctive feature of Mpox compared to other diseases (31, 32). The skin eruption usually begins within 1–3 days of the event of fever (11, 30). The rash tends to be concentrated more on the face and extremities rather than on the trunk. The following body areas are mainly affected: the face (95%); palms of the hands and soles of the feet (75%); oral mucous membranes (70%); genitalia (30%); and conjunctivae (20%) (32). The rash evolves sequentially from macules to papules, vesicles, pustules, and crusts, which dry up and fall off. The number of lesions varies from a few to several thousand. Complications include secondary infections, bronchopneumonia, sepsis, and encephalitis (13).

### 1.3. Diagnosis and therapy

Polymerase chain reaction (PCR) is the gold standard laboratory test for the confirmation of Mpox. Optimal diagnostic samples for Mpox are from skin lesions, fluid from vesicles and pustules, and dry crusts. Monkeypox virus has been demonstrated to be highly prevalent in seminal specimens, detectable early from day 1 and up to 19 days after symptoms onset and corroborating the role of potential sexual transmission of the disease (33). Clinical care for Mpox should be fully optimized to alleviate symptoms, manage complications, and prevent long-term sequelae. Patients should be offered fluids and food to maintain adequate nutritional status. Most cases can remain at home with supportive care (34).

### 1.4. Vaccination and prevention

Early recognition of cases, identifying risk factors, doing appropriate active contact tracing, and strengthening surveillance are the best defenses against a worldwide spread and are fundamental to containing a Mpox outbreak. Therefore, the education of healthcare workers and patients is of primary importance (13, 35). Infected patients should remain isolated until their rash is completely cured; abstention from sexual activity and close physical contact is also strictly advised until the rash heals (23).

Vaccination against smallpox was demonstrated to be ~85% effective in preventing Mpox, resulting in milder illness (36). However, we must take into account that access to vaccine supplies varies significantly from one state to another, based on several factors, such as macroeconomic situation, vaccine production capacity, resources, and health policies. At the beginning of the epidemic, supplies in Europe and the United States were low, but they were quickly implemented. Instead, even now, African and South American continents still have difficulty supplying vaccines (37–40). Moreover, these are non-replicating live attenuated third-generation vaccines, which were previously approved for protection against smallpox and extended to include protection against Mpox and related orthopoxvirus (21, 41). The European Medicines Agency (EMA) has recommended the vaccination for healthcare workers who are caring for or who may need to treat a patient with confirmed Mpox (29), gay, bisexual, transgender, and other men who have sex with men at the highest risk of exposure but also all the people with risk behaviors (such as people who have multiple partners, people who are used to mix sexual acts, and the use of drugs or to participate in the group).

## 2. Case report

The first confirmed case in Italy was dated 20 May 2022, which was identified by the National Institute of Infectious Diseases Spallanzani in Rome. Since that day, the Italian Ministry of Health has activated a health surveillance system with Italian Regions and has published a bulletin every Tuesday and Friday. On 15 July 2022, a total of 339 cases were confirmed in Italy, of which 337 were men with an average age of 37 years (20–71 years), and infection in 107 of them was related to recent trips abroad (42). As of 17 November 2022, the confirmed cases in Italy have increased to 957, of which 943 were men with an average age of 37 years (14–71 years) and 253 were reported to be in contact with foreign travelers (43).

With regard to the Veneto region of Northeast Italy, where the cases are reported and treated, so far, 66 cases of Mpox have been found (43); the clinical case under discussion is certainly the first regional case diagnosed and almost certainly the starting case of the Veneto region outbreak.

**Figure 1:**
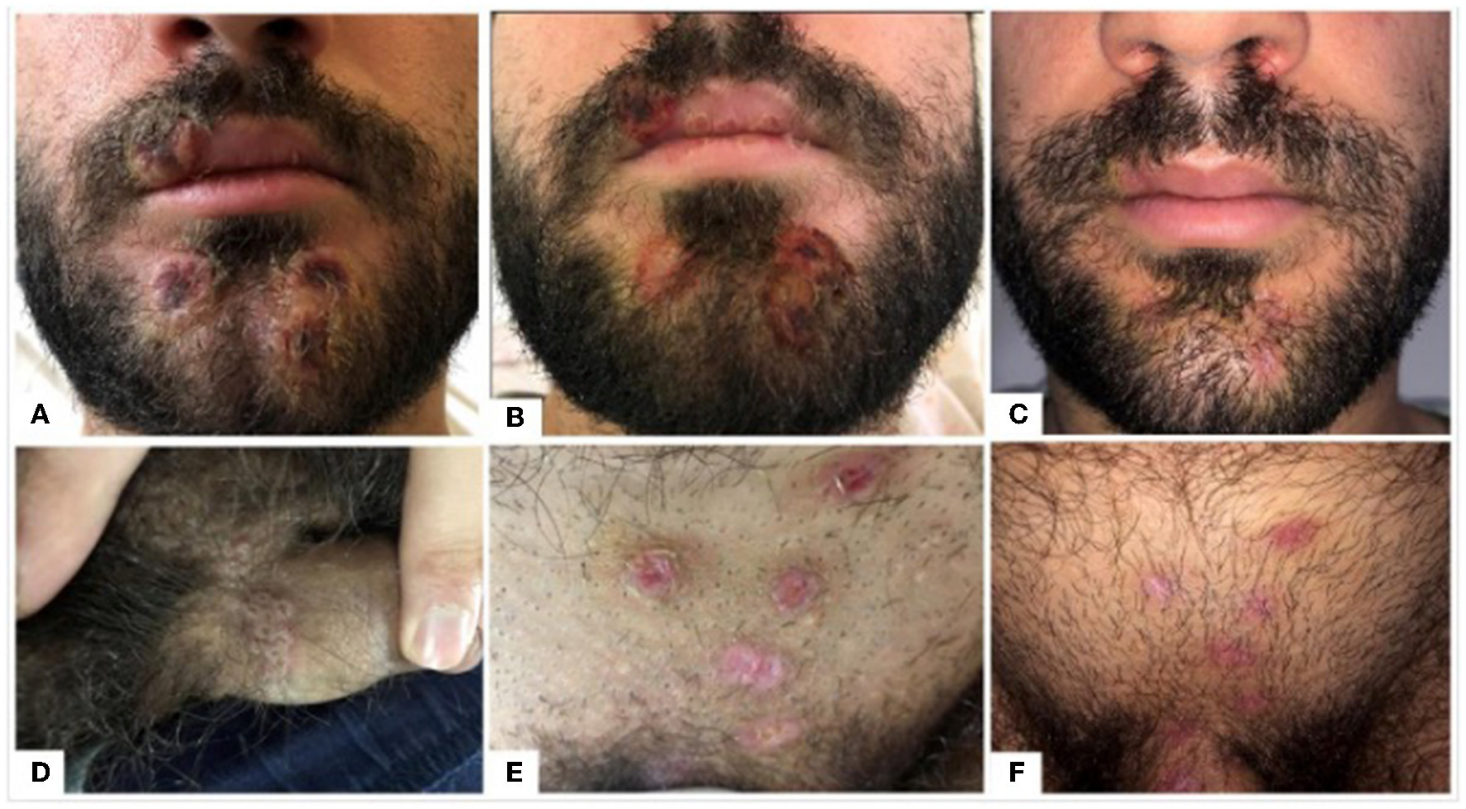
**(A–F)** Appearance and evolution of genital and facial lesions caused by MPXV in a young male patient. **(A–C)** The evolution of the facial lesions from the classic mouth sores **(A)** to a wide crusty ulcer **(B)** to finally a suitable resolution after ~3 weeks of using a chlortetracycline hydrochloride ointment twice a day **(C)**. **(D–F)** The evolution of the genital lesions from smaller umbilicate grouped lesions **(D)** to a broader skin ulcer **(E)** to healed and reddish lesions **(F)** after the topical treatment with chlortetracycline hydrochloride ointment.

A healthy 30-year-old man came to our clinic in June 2022 for the presence of a facial and genital rush (Figures 1A, D) with a fever of >39°C. The anamnesis was positive for those who were in contact with people who came from Spain 7 days ago. The facial lesions developed after the genital lesions and the onset of fever. They were characterized by a well-circumscribed and firm round shape with raised edges and crusty umbilication at the center (the so-called canker sores). The lesions were mostly asymptomatic. The patient did not experience any other systemic symptoms apart from fever and locoregional lymphadenopathy, which is a distinctive feature of Mpox compared to other rash illnesses, such as chickenpox, measles, varicella zoster, herpes zoster, herpes simplex, scabies, syphilis, and medication-associated allergies.

Due to the clinical and anamnestic data, the patient was admitted to the Infective Disease Unit, where a PCR test was performed on samples of the skin lesions, confirming the diagnosis of Mpox virus infection.

**Figure 2:**
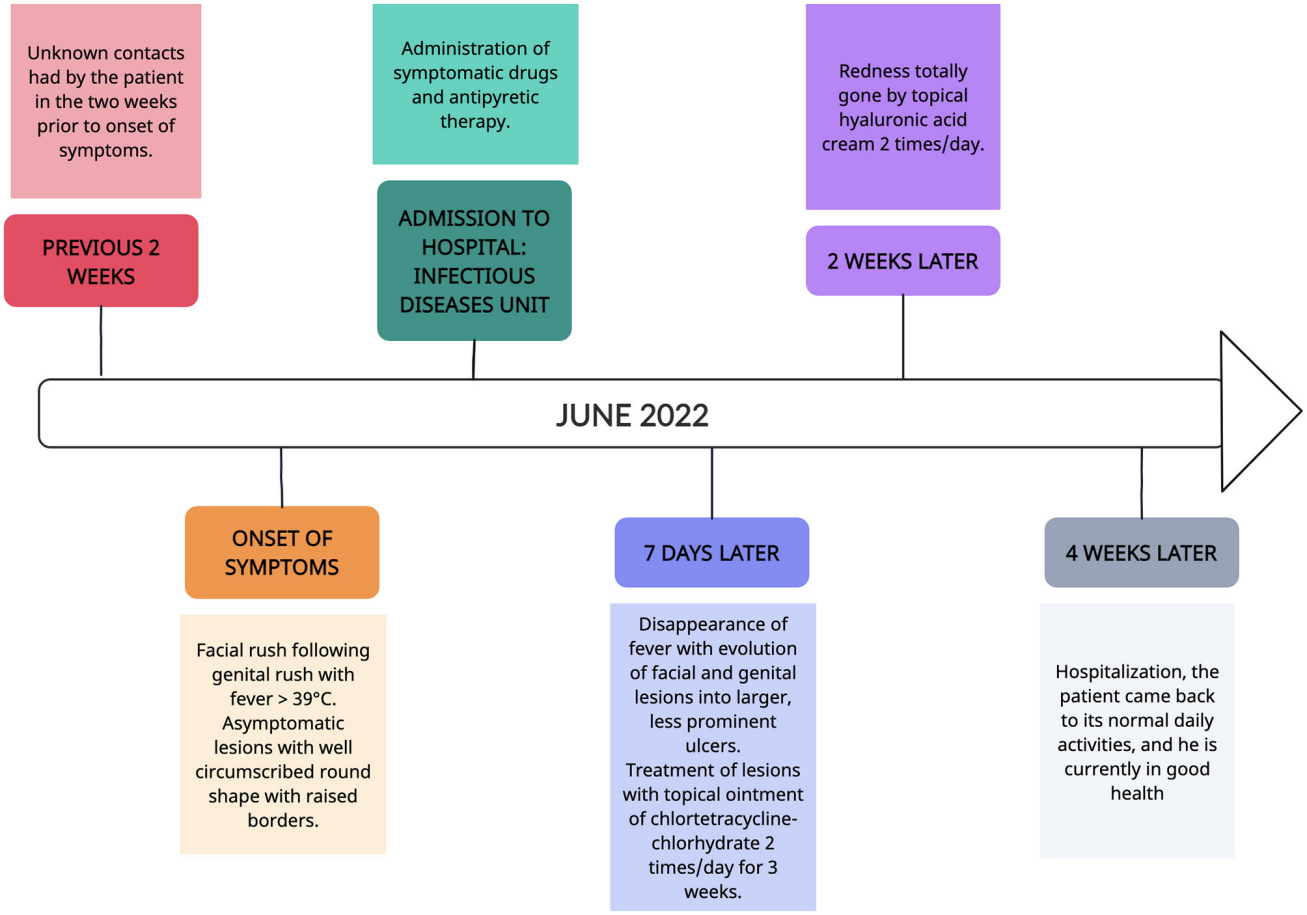
Timeline of the episode of patient care.

During the stay in the hospital, the patient was given just symptomatic therapy to reduce fever (paracetamol and NSAIDs), with its complete remission after 7 days. The facial and genital lesions evolved after 7 days into more extensive and less elevated crusty ulcers (Figures 1B, E), and they were treated with the application of topical chlortetracycline hydrochloride ointment twice a day for 3 weeks with an excellent remission and cosmetic outcome, without particularly evident scarring (Figures 1C, F). The residual redness resolved in about 12 weeks using a topical cream-based hyaluronic acid twice a day. Furthermore, 4 weeks after the hospitalization, the patient came back to his normal daily activities and is currently in good health. The timeline of the episode of the care is shown in Figure 2. The patient did not choose to diffuse public information concerning the contagion chain and concerning possible behaviors with increased risks of expositions associated with the disease, such as if he had multiple sexual intercourses in the last 2 weeks before the illness breakout or the details on if he had multiple unprotected sexual intercourses. Multiple specialists assisted the patient and have been involved in this case report, highlighting the fundamental role of an interprofessional team in caring for patients with this disease and reporting the necessity of collaboration in diagnostic and therapeutic processes by minimizing possible outbreaks and disease risks.

## 3. Discussion

There are many hypotheses concerning the ways Mpox spread in non-endemic regions during the current outbreak, but the dominant theory is that it spread from the UK and Spain to an increased extent among men who have sex with men (MSM) (44–47).

This case report highlights the new challenges against Mpox, which currently appears to present differently from the previous classical description, with possible human-to-human transmission through sexual contact (5). These differences can also be deduced from the localization of lesions, mostly present in the genital and oral area, and by MPXV DNA identified in many biological samples different than skin lesions, such as semen and rectal swabs (48, 49). In fact, during the current epidemic, both the case report and the other literature articles noted few skin lesions, almost exclusively found in the genital, anal, and oral areas with an asynchronous pattern and with inguinal lymphadenopathy, which differed significantly from the clinical description of the disease developed in the 1970s (5).

Thus, health institutions must communicate precisely and scientifically the elements of change and possible preventive actions to stop the spread of the epidemic outbreak. Several recent studies have stressed the importance of implementing preventive strategies following the anomalous outbreak in non-endemic areas, including providing protective equipment to healthcare personnel (gloves, masks, and protective clothing), keeping apart infected people in isolated rooms, immunizing high-risk groups like healthcare workers, and encouraging hand hygiene (23, 44, 50).

Contact tracing for MPXV is necessary as it is considered an infectious disease that requires notification in most countries, such as Italy (51). However, tracing the chain of contagion is not an easy task. The transmission of diseases was ascribed to sexual contact in 95% of reported cases (although it was never confirmed) (52), and patients usually had sex with multiple, often anonymous, partners; these factors are fueling the stigma on stating the facts to health authorities. A similar difficulty can also be seen in the case report where the patient was unwilling to provide any information related to the contagion chain and demonstrated possible higher-risk exposure behaviors associated with the disease. This phenomenon is significantly related to the media's creation of a close correlation between MSM, Mpox, and HIV to gain public attention risking further stigmatization of Mpox carriers, as it happened during the HIV epidemic in the 1980s (53–55).

All these considerations led WHO and CDC to appeal to the scientific community to reduce the stigma against Mpox through appropriate public communication and community engagement (56, 57). On 28 November 2022, WHO decided to use a new term, “Mpox,” as a synonym for monkeypox. The decision to invite all countries to adopt the new term aims to battle against the spread of racist and stigmatizing language both online and in other settings and specific communities. Mpox will become a preferred term after a 1-year transition period. This serves to ease concerns raised by experts about the confusion caused by a name change during a global outbreak (58).

Aware of the need to intensify surveillance and communication in specific population groups, such as the MSM community during the current outbreak (44), it is equally important to disseminate precise communication to the entire population that must not be directed only to high-risk groups, i.e., MSM, people living with HIV/AIDS (PLWHA), and the lesbian, gay, bisexual, transgender, and queer (or questioning), plus other sexual and gender identities (LGBTQI+ community) (59). Communication strategies must therefore be offered to involve a heterogeneous public based on the different risks of exposure but without stigmatizing attitudes (55). The need to involve a large and diverse audience is also demonstrated by the identification of the virus in people considered “anomalous cases” in the current epidemic panorama, such as a Belgian child without risk factors or in girls/women, as in Italy, where, in August 2022, 1% of MPXV infections were detected (60, 61). As a result, as our health policy regarding Mpox is currently primarily directed at specific groups of patients, namely, the MSM and STD patients, such communication must also be transparent and free from judgment and stigma. This is because the aforementioned groups often face major obstacles in accessing health services and vaccination due to the fear of judgment and discrimination they may face (38). Public health policies must learn from the lessons of previous outbreaks and take corrective actions to limit the recent spread of the anomalous MPXV outbreak (46, 50). Ideally, the health policy response to epidemics must be guided by three core principles, namely, equity, the inclusion of the most vulnerable, and community participation, as a solution to the epidemic, but must not be guided as a cause of epidemic (38, 40).

## 4. Conclusion

To prevent the growing spread of this disease and to effectively control this anomalous spread, early diagnosis, isolation, effective contact tracing, and targeted vaccination strategies are critical. Properly implementing all response measures requires strong risk communication and community engagement, as well as the involvement of even the most vulnerable and socially disadvantaged groups (40). There must be a greater awareness and preparation of health workers to raise vaccination adherence and reduce fear and discrimination in access to health care for these specific people.

Stigma represents a barrier to the involvement of adequate care and the provision of essential information for contact tracing. A revolutionary approach is communicating this event as a “clustering of infected cases in certain high-risk groups,” identifying specific risk factors through in-depth and evidence-based epidemiological investigations (54, 55). The first step is gradually changing the name of the disease to deprive it of the negative and stigmatizing connotation it has now assumed. Finally, anomalous outbreaks in non-endemic countries, with no causal links identified, must become an alarm signal for governments and health policies to start designing national preventive plans for the management of unexpected outbreaks—as is happening in Italy—following the severe acute respiratory syndrome coronavirus 2 (SARS-CoV-2) pandemic, for the future flu epidemics (62).

## Data availability statement

The original contributions presented in the study are included in the article/supplementary material, further inquiries can be directed to the corresponding author.

## Ethics statement

The patient provided written informed consent to publish this manuscript, photos, and for material sampling. Photos have been anonymized. Written informed consent was obtained from the participant/patient(s) for the publication of this case report.

## Author contributions

Conceptualization, methodology, writing—original draft, writing—review, and editing: GG, MB, RG, and TB. Data curation: GG, MB, RG, TB, and MA. Cared for the patient: MB, GG, and MA. Visualization: GG, RG, and TB. Supervision: TB and MA. All authors have read and agreed to the final approval of the version to be submitted.
